# Mass drug administration of antibacterials: weighing the evidence regarding benefits and risks

**DOI:** 10.1186/s40249-022-00998-6

**Published:** 2022-06-30

**Authors:** Robert J. Rolfe, Hassaan Shaikh, L. Gayani Tillekeratne

**Affiliations:** 1 Division of Infectious Diseases, Department of Medicine, Duke University School of Medicine, Durham, NC USA; 2 Duke Global Health Institute, Duke University, Durham, NC USA; 3 Department of Medicine, University of Pittsburgh Medical Center, McKeesport, PA USA; 4 Department of Medicine, Faculty of Medicine, University of Ruhuna, Galle, Sri Lanka

**Keywords:** Mass drug administration, Child mortality, Under-developed nations, Antibacterial drug resistance

## Abstract

**Background:**

Mass drug administration (MDA) is a strategy to improve health at the population level through widespread delivery of medicine in a community. We surveyed the literature to summarize the benefits and potential risks associated with MDA of antibacterials, focusing predominantly on azithromycin as it has the greatest evidence base.

**Main body:**

High-quality evidence from randomized controlled trials (RCTs) indicate that MDA-azithromycin is effective in reducing the prevalence of infection due to yaws and trachoma. In addition, RCTs suggest that MDA-azithromycin reduces under-five mortality in certain low-resource settings that have high childhood mortality rates at baseline. This reduction in mortality appears to be sustained over time with twice-yearly MDA-azithromycin, with the greatest effect observed in children < 1 year of age. In addition, observational data suggest that infections such as skin and soft tissue infections, rheumatic heart disease, acute respiratory illness, diarrheal illness, and malaria may all be treated by azithromycin and thus incidentally impacted by MDA-azithromycin. However, the mechanism by which MDA-azithromycin reduces childhood mortality remains unclear. Verbal autopsies performed in MDA-azithromycin childhood mortality studies have produced conflicting data and are underpowered to answer this question. In addition to benefits, there are several important risks associated with MDA-azithromycin. Direct adverse effects potentially resulting from MDA-azithromycin include gastrointestinal side effects, idiopathic hypertrophic pyloric stenosis, cardiovascular side effects, and increase in chronic diseases such as asthma and obesity. Antibacterial resistance is also a risk associated with MDA-azithromycin and has been reported for both gram-positive and enteric organisms. Further, there is the risk for cross-resistance with other antibacterial agents, especially clindamycin.

**Conclusions:**

Evidence shows that MDA-azithromycin programs may be beneficial for reducing trachoma, yaws, and mortality in children < 5 years of age in certain under-resourced settings. However, there are significant potential risks that need to be considered when deciding how, when, and where to implement these programs. Robust systems to monitor benefits as well as adverse effects and antibacterial resistance are warranted in communities where MDA-azithromycin programs are implemented.

**Graphical Abstract:**

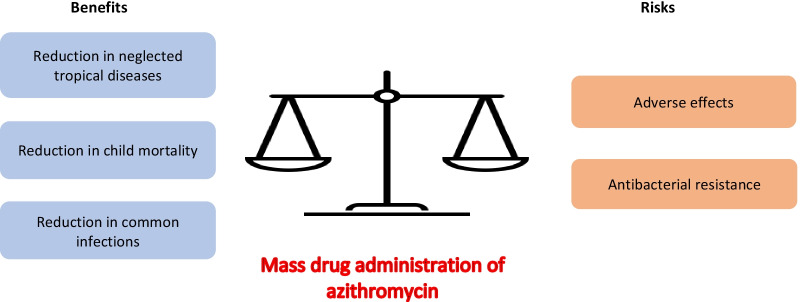

## Background

Mass drug administration (MDA) is a strategy to improve health at the population level through the widespread delivery of safe and inexpensive medications for the prevention and treatment of disease [1]. Although unusual in high-income settings, MDA remains relevant in resource-limited settings, as it does not require individual diagnosis or treatment decisions [1–4].

MDA programs have targeted both infectious and non-infectious threats, ranging from programs distributing anti-parasitic agents for decreasing malaria incidence to programs distributing vitamin A supplements for reducing childhood blindness [5]. However, it is in battling the neglected tropical diseases (NTDs), a group of 20 diseases that typically affect the world’s poorest citizens, that MDA has met with the greatest success [1]. MDA has been associated with a substantial reduction in the burden of the infectious diseases targeted by these campaigns, including trachoma, onchocerciasis, lymphatic filariasis, soil-transmitted helminthic infections, and schistosomiasis [6]. In 2017, at least 1.7 billion MDA treatments with antimicrobials were delivered to 1 billion people for the prevention and treatment of NTDs [7]. Recently, MDA programs distributing antibacterials have gained increased attention due to evidence that they may reduce childhood mortality [5, 8, 9]. This use of MDA for reduction of childhood mortality is in contrast to the prior use of antimicrobials in MDA campaigns, when a specific pathogen was targeted.

To date, most MDA programs using an antibacterial agent have distributed oral azithromycin, a macrolide antibacterial that has a long half-life (67 h) and high tissue penetration [10]. Other antibacterials such as intramuscular (IM) benzathine penicillin G and topical tetracycline have also been used in MDA programs, but to a much lesser extent. Benzathine penicillin G is a beta-lactam antibacterial that can be used as a single-dose, IM treatment for yaws, but it is being replaced by oral azithromycin given the ease of delivery associated with the latter [11]. Tetracycline can be used in topical form for the treatment of trachoma, but it is predominantly used in children < 6 months of age in whom the safety of azithromycin is debated, as topical therapy must be administered twice daily for 6 weeks [12].

Since MDA programs distributing antibacterials have largely used oral, single-dose azithromycin, this review will focus on the literature on MDA-azithromycin. We survey the evidence regarding benefits and risks associated with MDA-azithromycin programs and raise questions that are critical in determining the future of these programs, specifically when used to reduce childhood mortality.

## Methods

### Results

Data for this review were initially identified through a search of PubMed using the search terms “mass drug administration” and “antibiotic.” This search provided an initial set of studies relevant to the topic. References from the initially identified studies were also reviewed and included in this review if relevant. Only articles published in English were included, and articles were not limited based on year of publication. Only peer-reviewed, published studies were considered for this review.

### MDA-azithromycin programs: diseases and conditions for which there is probable benefit

Existing evidence and World Health Organization (WHO) recommendations support the use of MDA-azithromycin in treating two NTDs, trachoma and yaws, and in reducing under-five mortality in regions most affected by these conditions [2, 13, 14]. All three conditions are associated with poverty, crowded households, and lack of access to water and sanitation. In addition, children < 10 years of age are the main target of all three MDA programs.

#### Trachoma

MDA-azithromycin first gained widespread use in the treatment of trachoma, the most common infectious cause of blindness. Approximately 21 million people worldwide have active trachoma and 232 million people live in trachoma-endemic areas worldwide [13]. Trachoma is caused by certain serovars of *Chlamydia trachomatis*, with repeated episodes of ophthalmic infection and conjunctival inflammation, known as active trachoma, causing scarring of the eyelids and eventually leading to corneal opacification and blindness [15]. Children are the core group for transmission, and in endemic areas, active trachoma may affect up to 60–90% of preschool children [2, 16].

In 1998, the World Health Assembly endorsed the Alliance for the Global Elimination of Trachoma by 2020 (GET2020) and recommended the SAFE strategy for eliminating trachoma (Surgery, Antimicrobial distribution, Facial cleanliness, and Environmental improvements) [17]. Annual, district-wide MDA of azithromycin was recommended as part of this strategy for districts with a prevalence of active trachoma ≥ 10% in children aged 1‒9 years of age [2]. To date, more than 900 million doses of oral azithromycin have been distributed through trachoma control programs [18].

High-quality studies support the use of MDA-azithromycin in trachoma control. A recent Cochrane review assessed four cluster randomized trials (RCTs) that compared community-level treatment with MDA-azithromycin and no treatment. Three of four studies showed a reduction of 40‒60% in ocular infection or active trachoma by 12 months. The fourth, lower-quality study did not show a difference in prevalence of active trachoma at 12 months in low-prevalence communities [12].

#### Yaws

Yaws is a relapsing non-venereal treponemal disease caused by *Treponema pallidum* subspecies *pertenue* and affects skin, bone, joints, and cartilage. From 2010 to 2013, 256,343 cases of yaws were reported to the WHO from 13 endemic countries, and there were an estimated 89 million people living in yaws-endemic districts in 2012 [19]. Approximately 75–80% of those affected by yaws are children less than 15 years of age, with peak incidence at 6‒10 years of age [20].

Previously, a single dose of IM, long-acting, benzathine penicillin G was recommended for MDA treatment of yaws. However, treatment with IM penicillin is resource intensive and can be painful. In 2011‒2012, two open-label, randomized controlled trials were conducted in yaws-endemic regions in Ghana and Papua New Guinea to explore the impact of treatment with oral azithromycin compared to IM benzathine penicillin [21, 22]. Both studies consisted of children < 15 years of age who had active yaws lesions. Treatment with a single dose of azithromycin was found to be non-inferior to a single dose of IM penicillin [21, 22]. These two studies set the stage for recommending MDA-azithromycin in the control of yaws.

#### Childhood mortality

The potential for MDA-azithromycin to decrease childhood mortality has gained a great deal of attention in recent years. MDA-azithromycin has been promoted as a strategy that could help the world meet targets such as Sustainable Development Goal (SDG) 3, which aims to eliminate all preventable deaths in children < 5 years of age and to reduce under-five mortality to at least 25 deaths per 1000 live births by 2030 [23]. In 2019, there were 5.2 million deaths in children under five years of age, with mortality highest in sub-Saharan Africa at 76 deaths per 1000 live births [24]. To date, there are 3 cluster randomized controlled trials (RCTs) that have assessed the impact of community level MDA-azithromycin compared to placebo on childhood mortality, with 2 of 3 showing benefit (Table 1). In addition, one cluster RCT which compared annual versus twice-yearly MDA-azithromycin and a pre-post continuation study of a prior cluster RCT both suggest mortality benefit associated with MDA-azithromycin. However, the underlying mechanism by which childhood mortality is decreased in these studies is unclear.

**Table 1:** Cluster randomized controlled trials assessing the impact of MDA-azithromycin on childhood mortality

Study	Countries and duration	Intervention and control arms	Study population	Medications used	Primary outcome	Findings
Trachoma Amelioration in Northern Amhara (TANA) [5]	Ethiopia, 2006‒2007	(1) Annual azithromycin distribution, (2) twice-yearly azithromycin distribution, (3) quarterly azithromycin distribution, and (4) a control group for whom treatment was delayed for 12 months	Arms 1, 2, and 4 included anyone 1 year of age or older, Arm 3 included children 1–9 years of age	Adults were treated with 1 g of azithromycin and children were treated with 20 mg/kg of azithromycin (maximum 1 g)	Mortality in children 1‒9 years of age, as measured at approximately 12 months after initial dosing	49% reduction in death in children aged 1‒9 years (pooled across all treatment arms), compared to placebo
The Partnership for the Rapid Elimination of Trachoma (PRET) [16]	Niger, 2010‒2013	(1) Annual azithromycin distribution, and (2) twice-yearly azithromycin distribution	Arm 1 included the entire community and arm 2 included children 0‒12 years of age	All participants ≥ 6 months of age received 20 mg/kg oral azithromycin (maximum 1 g) and children < 6 months of age, pregnant women, and those allergic to macrolides were offered topical tetracycline ointment (1%) for 6 weeks	Prevalence of ocular chlamydial infection in children aged 0‒5 years, as monitored by polymerase chain reaction at 36 months	Mortality rate was 35.6 deaths per 1000 person-years in the annual arm and 29.0 deaths per 1000 person-years in the twice-yearly arm. The mortality rate ratio comparing children in the twice-yearly arm to the annual arm was 0.81 (95% *CI*: 0.66‒1.00) [27]
The addition of azithromycin to seasonal malaria chemoprevention [26]	Burkina Faso and Mali, 2014‒2017	(1) Distribution of azithromycin together with sulfadoxine-pyrimethamine plus amodiaquine in four 3-day cycles at monthly intervals for three successive seasons, and (2) a control group receiving a placebo instead of azithromycin with the same combination and schedule	Both arms included children 3 to 59 months of age	Infants 3–11 months of age received a combined 250 mg of sulfadoxine and 12.5 mg of pyrimethamine plus 75 mg of amodiaquine, along with either 100 mg of azithromycin or a matching placebo. Children 1–4 years of age received double these doses	Death or hospital admission for at least 24 h that was not due to trauma or elective surgery	The addition of azithromycin to the antimalarial drugs for chemoprevention did not result in a lower incidence of death or hospital admission in comparison to the placebo group
MORDOR I (Macrolides Oraux pour Réduire les Décès avec un Oeil sur la Résistance) [8]	Malawi, Niger, and Tanzania, 2014‒2017	(1) Twice-yearly azithromycin distribution, and (2) a control group receiving twice-yearly placebo	Both arms included children 1–59 months of age	Oral azithromycin 20 mg per kilogram of body weight	Aggregate all-cause mortality in study population	Mortality was 13.5% lower overall in communities that received azithromycin. Children in the age group of 1–5 months had the greatest effect from azithromycin (24.9% lower mortality)
MORDOR II [9]* (Macrolides Oraux pour Réduire les Décès avec un Oeil sur la Résistance)	Niger, 2017‒2018	The Niger component of the MORDOR I trial in which children 1–59 months old in communities were assigned to four twice-yearly distributions of azithromycin or placebo were all given two additional open-label azithromycin distributions during two additional twice-yearly distributions	Children 1–59 months of age	Oral azithromycin 20 mg per kilogram of body weight	Aggregate all-cause mortality in study population	In communities that had originally received placebo, mortality decreased by 13.3% when the communities received azithromycin. No significant difference was noted in the communities that had originally received azithromycin

^*^ Continuation of a prior cluster randomized controlled trial

The five studies were conducted in six countries in which the under-five mortality rate is high: Ethiopia; Malawi, Niger, Tanzania, Mali, and Burkina Faso [8, 9, 16, 25, 26]. All five studies carried out randomization at the community level. Of the three placebo-controlled trials, the first study (TANA) was conducted in Ethiopia from 2005 to 2006, the second was conducted in Mali and Burkina Faso from 2014 to 2016, and the third (MORDOR I) was conducted in Malawi, Niger, and Tanzania from 2014 to 2017. The three studies implemented MDA-azithromycin in children aged 1 year and older and adults, children 3‒60 months of age, and children 1‒59 months of age, respectively. Table 1 details the dosing and schedule of the MDA intervention. All trials studied azithromycin monotherapy in the intervention arm, except for the second study, which included azithromycin superimposed on seasonal malaria chemoprophylaxis (SMC) with sulfadoxine-pyrimethamine and amodiaquine in both study arms. TANA showed an almost 50% reduction in mortality 12 months after the intervention in children 1‒5 years of age. The second study did not show a reduction in mortality in children aged 3‒59 months over a period of 3 years (as per data obtained by the WHO) [2]. MORDOR I showed an almost 15% reduction in mortality 26 months after intervention in children < 5 years of age. Sub-group analysis of MORDOR I showed that the reduction in mortality was significant only in Niger but not in the other two countries. In addition, children between 1 and 5 months of age had the highest overall mortality, but this age group also had the largest difference in mortality rates when comparing those in the treatment and placebo arms with 24.9% lower mortality. However, effect modification by age was not statistically significant [8].

In addition to these 3 cluster RCTs, there was one cluster RCT (PRET) conducted in Niger from 2010 to 2013 that compared annual treatment for all individuals in the community over 6 months of age with twice-yearly treatment in persons 6 months‒12 years [16, 27]. PRET showed a reduction in mortality of 20% in twice-yearly treatment compared to annual treatment. Another longitudinal study (MORDOR II, continuation of MORDOR I) conducted in Niger from 2017 to 2018 compared two additional twice-yearly doses of azithromycin in groups that had previously received placebo. MORDOR II showed a mortality reduction of 13.3% compared to prior to the intervention, and also showed that the mortality reduction in the group that originally received azithromycin-MDA was sustained [9]. Finally, more recently, a multi-site RCT in Kenya among children aged 1‒59 months who were randomized to receive a five day course of azithromycin or placebo after discharge from hospital admission showed no statistical difference in death or rehospitalization in the six months following hospital discharge [28]. However, this study was not an MDA-azithromycin study.

Following the publication of results from MORDOR I, the WHO released guidelines in 2020 regarding the use of MDA-azithromycin to reduce childhood mortality. They recommended against universal implementation of MDA-azithromycin for prevention of childhood mortality, and that MDA-azithromycin be considered in children 1‒11 months of age for prevention of childhood mortality in sub-Saharan settings where (1) infant mortality is > 60 per 1000 live births or under-five mortality is > 80 per 1000 live births; (2) infant and under-five mortality rates, adverse effects, and antibiotic resistance are continuously monitored; and (3) existing child survival interventions, such as SMC where recommended, is concurrently strengthened. The recommended dose of azithromycin was 20 mg/kg every 6 months. The committee stated that the guideline would be applicable for 2‒3 years, at which time new data were expected [2].

Since the release of the WHO guidelines, a secondary analysis from MORDOR-Malawi has shown that MDA-azithromycin has the potential to be very cost effective, but that wide geographic variation in effectiveness exists [29]. Another study has projected that a targeted strategy, in which high-risk children are selected for receipt of azithromycin, has higher cost effectiveness compared to MDA-azithromycin and would minimize azithromycin exposure in the community [30]. Further studies regarding the effectiveness and cost effectiveness of MDA-azithromycin in reducing childhood mortality have not been published.

### What are the potential mechanisms by which MDA-azithromycin may reduce mortality?

Despite apparent success in reducing childhood mortality, the mechanism by which MDA-azithromycin reduces childhood mortality has not yet been elucidated. Understanding how MDA azithromycin reduces childhood mortality may help determine which settings and populations should be targeted for implementation. It has been hypothesized that MDA-azithromycin may reduce deaths from respiratory infection, diarrheal infection, and/or malaria in treated children and their close contacts, since these illnesses are major causes of childhood mortality in low- or middle-income countries (LMICs) [6]. Impact on inflammation or chronic disease is also possible, since macrolides are rare among antibacterials as they may be used to combat chronic lung disease [31]. MORDOR I suggested that the greatest mortality benefit was in children < 1 year of age, thus conditions affecting children in this age group may particularly shed light. Table 2 details infections for which azithromycin is commonly used in the clinical setting. Treatment of these infections may contribute to the reduction in childhood mortality.

**Table 2:** Common infectious syndromes or pathogens that may be treated with azithromycin in the clinical setting

Syndrome or specific pathogen	Age group mostly affected	Serious consequences of untreated Infection	Evidence or guidelines supporting azithromycin use	Evidence from MDA studies supporting or refuting use of azithromycin for syndrome/ pathogen
Skin and soft tissue infection/ impetigo, most commonly caused by *Staphylococcus aureus* and *Streptococcus pyogenes*. Can occur as a secondary bacterial infection following scabies infection, a parasitic NTD for which MDA of the anti-parasitic ivermectin has been used	Children and adults	Bacteremia, glomerulonephritis, and potentially rheumatic heart disease	Macrolides can be used in the treatment of impetigo. The IDSA recommends erythromycin as an alternative to beta lactam-based therapies [70]	•In the Solomon Islands, a cluster RCT of ivermectin MDA only or ivermectin co-administered with azithromycin to all residents showed a similar decrease in impetigo prevalence in both arms at 12 months [57] •Ivermectin plus either oral azithromycin or topical tetracycline showed 74% reduction in impetigo prevalence at 12 months in azithromycin arm in the Solomon Islands [71]. Three years after the intervention, the prevalence of scabies had decreased by 74.9% and the prevalence of impetigo had decreased by 61.3% [72] •Among children £10 years in Nepal, MDA-azithromycin was associated with a 60% decrease in impetigo 10 days after treatment, but levels returned to baseline by 6 months [73]
Rheumatic heart disease caused by group A *Streptococcus*	Children	Long term disability or death	IDSA recommends azithromycin as an alternative to penicillin in treatment for group A streptococcal pharyngitis and secondary prophylaxis of acute rheumatic disease [74] Erythromycin is an alternative for penicillin-allergic patients for primary and secondary prophylaxis of rheumatic heart disease, but it should not be used in areas where *S. pyogenes* has high rates of macrolide resistance [75]	•The reduction of rheumatic heart disease has not been reported in the literature on MDA-azithromycin programs
Acute respiratory Infection	Children and geriatric populations	Respiratory failure or death	Single-dose azithromycin 1500 mg reduced risk of community-acquired pneumonia by 50% compared to no therapy in male Russian military recruits (10.3% developed pneumonia compared to 20.2% in the control group over 22 weeks) [76]	•The incidence of upper respiratory infection over three malaria seasons was 15% lower in children treated with MDA antimalarials and azithromycin compared to children treated with MDA antimalarials and placebo in a cluster RCT in Burkina Faso and Mali [26] •In a cohort of children < 5 years in Tanzania, a single round of MDA-azithromycin was associated with a short-term (1‒3 months after administration) decrease in ARI of 38% compared to untreated communities, but this difference was not sustained after 1 month [77] •In a cohort of children £10 years in Nepal, ARI symptoms did not decrease after MDA-azithromycin [73] •One cluster RCT that examined MDA-azithromycin in the Gambia among children < 14 years treated with 3 doses of azithromycin did not show a reduction in ARI following MDA-azithromycin [78]
Diarrheal illness	Children	Stunting, vitamin deficiencies, death	Azithromycin is one of the first-line agents for the treatment of *Campylobacter* spp, *Shigella* spp, non-typhoidal *Salmonella* spp, and enteric fever due to *Salmonella typhi* or *Salmonella paratyphi* [55]	•In a cluster RCT in the Gambia, there was an almost 50% reduction in diarrhea at 28 days in children < 14 years treated with 3 doses of azithromycin [78] •In a cluster RCT in Mali and Burkina Faso, there was a 15% reduction in diarrhea in the azithromycin-MDA arm compared to placebo arm, when given with SMC [26] •In a cohort study in Nepal, there was a 75% reduction in diarrheal illness following one round of MDA-azithromycin in children ≤ 10 years [73] •In a cohort study in Tanzania, there was no significant reduction in diarrheal illness among children < 5 years of age after one round of MDA-azithromycin.[79]
Malaria	Children and to a lesser extent adults in endemic areas		Azithromycin displays weak antimalarial activity [80]	•In a cohort of children < 5 years in Tanzania, a single round of MDA-azithromycin was associated with a short-term (only in first month), 73% reduction in *Plasmodium falciparum* infections in treatment versus control villages [81] •Cluster RCT in Mali and Burkina Faso of MDA-azithromycin versus placebo with malaria SMC showed no decrease in laboratory-confirmed malaria in children < 5 years of age [26]
Syphilis	Adults	Cardiovascular disease, tabes dorsalis	In the setting of a penicillin allergy, azithromycin is recommended by the WHO as an option for early syphilis treatment if local susceptibility is likely and 14 days of doxycycline cannot be used [82]	•In Rakai, Uganda, in a sub-analysis of a cluster-randomized trial assessing STD control, participants received either single-dose benzathine penicillin G, single-dose azithromycin 1 g, or combination of the two drugs for participants with positive syphilis serology. There was no difference in cure rates of syphilis by treatment group [83]
Chlamydia	Adults	Ectopic pregnancies, pelvic inflammatory disease, infertility	Azithromycin is recommended for treatment of chlamydia [84]	•A study of gonorrhea and chlamydia rates in the Solomon Islands after an MDA-azithromycin campaign for trachoma showed a 40% reduction in the age-adjusted prevalence of *C. trachomatis*. Gonorrhea rates did not change [85]

*NTD* neglected tropical disease, *MDA* mass drug administration, *RCT* randomized controlled trial, *IDSA* Infectious Diseases Society of America, *WHO* World Health Organization

Verbal autopsies were conducted in the MDA-azithromycin childhood mortality studies to determine causes of death and may shed light on the mechanism by which MDA-azithromycin reduces mortality. Table 3 summarizes these verbal autopsy data. However, even these data to date are conflicting, and sub-studies have been under-powered to answer the question accurately. Some studies show that common illnesses such as respiratory illnesses, diarrheal illnesses, and malaria were less common in the MDA-azithromycin groups, while others do not show such differences. In the Niger arm of MORDOR I, which was the definitive study showing mortality benefit with MDA-azithromycin in children, the proportions for individual types of infections did not vary significantly between treatment and control arms [32]. If reduction in one type of infection were driving the change in mortality, we would expect the proportion of deaths due to this infection to be decreased in the treatment compared to placebo arms.

**Table 3:** Verbal autopsy data on infections in MDA of azithromycin to reduce childhood mortality studies

Study	Outcome
TANA	No statistically significant difference in cause of death in children aged 1‒9 was seen between the treated and untreated groups through verbal autopsy of 55 children with documented cause of death [5]
Two additional clusters in the TANA study (not reported in original study)	Treatment was associated with a 65% reduction in all-cause mortality and 80% reduction in infectious mortality (malaria, respiratory infection, diarrhea) in children 1‒5 years of age [25]
Azithromycin or placebo added to sulfadoxine-pyrimethamine plus amodiaquine during malaria-transmission season in Burkina Faso and Mali	No difference in mortality was seen between arms, but certain infections occurred with lower frequency in the azithromycin arm: gastrointestinal infections (15% lower), upper respiratory tract infections (15% lower), and nonmalarial febrile illnesses (20% lower) [26]
Random sample of 250 verbal autopsies from each site in MORDOR I	41% of deaths were due to malaria, 18% were due to diarrhea, and 12% were due to pneumonia. Causes of death differed significantly by site, with more deaths attributed to malaria in Niger and more to pneumonia in Tanzania [8]
Full verbal autopsies from Niger in MORDOR I	In the treatment group, 27.9% of deaths were due to malaria, 16.1% were due to pneumonia, and 14.9% were due to diarrhea. Similar numbers were seen in the control group Mortality in communities that received azithromycin was about 20% lower for malaria and pneumonia, and 30% lower for dysentery and meningitis The distribution of causes of death between the two groups did not differ significantly [32]
MORDOR I verbal autopsies in Malawi	Using two automated programs to conduct verbal autopsies, fewer HIV/AIDS deaths (30% less), pneumonia deaths (20‒40% less), and diarrheal deaths (30% less) were seen in the MDA-azithromycin arm compared to placebo [33, 86]
MORDOR I sub-study in Malawi*	Malaria parasitemia and gametocytemia did not change significantly between treatment and placebo groups at 12 and 24 months. Age and parasitemia were positively associated, suggesting that benefits may not be due to malaria [87]
Pooled data from TANA, PRET, and MORDOR I*	No convincing evidence that the baseline mortality rate modified the effect of azithromycin on mortality, suggesting that reduced infections may not have driven reduced mortality [88, 89]

*Analysis of clinical/ epidemiological data collected in the study and not a formal verbal autopsy study. *MDA* mass drug administration, *MORDOR* Macrolides Oraux pour Réduire les Décès avec un Oeil sur la Résistance, *PRET* the Partnership for Rapid Elimination of Trachoma, *TANA* Trachoma amelioration in Northern Amhara

Studies have also evaluated if the effect of MDA-azithromycin on childhood mortality may be related to anemia or nutritional status. A study conducted in parallel with MORDOR I was performed in 30 communities in Tanzania between 2015 and 2017. Children between the age of 1–59 months at baseline were eligible for inclusion and treated with twice-yearly azithromycin for 24 months. The outcome of interest for this study was mild moderate or severe anemia. Twice-yearly azithromycin treatment to preschool children did not significantly impact anemia prevalence in this study [33]. MDA-azithromycin did not impact growth or nutritional status in separate studies conducted in Niger, Ethiopia, the Gambia, Burkina Faso, and Mali [34–37].

### Can MDA-azithromycin offer ancillary benefit for conditions that are not explicitly targeted by MDA programs?

Macrolides are used commonly in the treatment of many community-acquired infections. The mass distribution of azithromycin has the potential to positively impact the burden of these infections. In Table 2, we summarized the data on the impact MDA-azithromycin may have on these common infections. These infections include skin and soft tissue infections/impetigo, rheumatic heart disease, acute respiratory infection, diarrheal illness, malaria, syphilis, and chlamydia. To date, studies assessing the impact of MDA-azithromcyin on these common infections have either not been conducted or have yielded mixed results. However, the potential for MDA-azithromycin to reduce the burden of these infections exists.

### What are the potential direct health risks associated with MDA-azithromycin?

Prior to the implementation of MDA-azithromycin campaigns on a large scale, it is important to understand the health risks that MDA-azithromycin may pose to the individual and the community, as well as which individuals may carry the greatest risk. The main direct health risks associated with azithromycin include gastrointestinal side effects, infantile hypertrophic pyloric stenosis (IHPS), cardiovascular side effects, and microbiome modulation which may contribute to the development of chronic diseases. In general, due to its broad antibacterial spectrum and safety profile, azithromycin is one of the most commonly prescribed antibacterials in children. In the last two decades, over 700 million doses of MDA-azithromycin have been delivered to children [38]. However, the safety of azithromycin use in infants is debated. There is a paucity of data regarding the safety of azithromycin in children < 6 months of age, and the WHO does not recommend use of azithromycin in this age group for the treatment of trachoma, although it does recommend azithromycin for use in children < 1 year for reducing childhood mortality [2]. Data regarding risks associated with azithromycin when administered in MDA programs are sparse.

#### Gastrointestinal side effects

Gastrointestinal side effects are among the most common adverse effects seen after the use of any antibacterial. In a study of almost 17,000 people in Ethiopia who received MDA-azithromycin, side effects were solicited three weeks after MDA. The prevalence of self-reported side effects was 9.6% among all age groups and 4.7% among children aged 1–9 years in the MDA-azithromycin arm. The most commonly reported side effects were abdominal pain (53.1%), nausea (21.7%), vomiting (12.8%), and diarrhea (12.5%). However, the prevalence of side effects in the placebo group was not reported [38, 39]. In a systematic review of neonates (< 28 days) who received at least one dose of azithromycin, data from 4 RCTs indicated that gastrointestinal side effects were seen in 19.6% of patients [40].

#### IHPS

IHPS is caused by thickening of the pylorus muscle in the stomach, with eventual gastric outlet obstruction that can cause vomiting and dehydration. It affects about 1.9 of every 1000 live births and is the most common indication for surgery in the first six months of life [41]. The risk of IHPS may be increased in infants taking oral erythromycin or azithromycin, especially during the first two weeks of life, due to the macrolides’ potent stimulation of gastrointestinal motility. In a retrospective cohort study from 2001 to 2012 of children in a US military database, exposure to azithromycin or erythromycin in the first 2 weeks of life was associated with 8 or 13 times greater odds of IHPS, respectively. There was no association between either antibacterial exposure and IHPS after 6 weeks of life through 12 weeks [42]. A meta-analysis found that infants exposed to erythromycin had an odds ratio of 2.45 for developing IHPS, and a 12-fold increase if they were given erythromycin in the first two weeks of life [41]. In a case series in Nigeria of 26 infants with IHPS, presentation for care and diagnosis was generally late, although many infants had symptoms that started in the neonatal period with a mean age at diagnosis of 7 weeks. Most infants received open pylorotomyotomy and 15% had post-operative complications. Three of the patients in this case series died, with two during resuscitation attempts prior to surgery and one during surgery [43].

As a follow up study to MORDOR, 30 communities that participated in the Niger arm were followed two weeks after treatment rounds to evaluate for adverse events. With each round of azithromycin, caregivers of infants aged 1‒5 months of age were surveyed regarding adverse events since treatment. There were no differences in the rates of adverse events between azithromycin and placebo, and notably no reported cases of IHPS in either arm [44]. Excluding infants less than one month of age in MDA azithromycin campaigns may decrease the risks of IHPS in infants.

#### Cardiovascular side effects

Several macrolides, including azithromycin, have been found to be proarrhythmic, with reports of QT prolongation, torsades de pointes, and ventricular tachycardia in the absence of QT prolongation. In the previously mentioned systematic review of adverse drug reactions to azithromycin in children, six prospective studies (five RCTs, one cohort study) reported 79 cardiac adverse events. QT prolongation was reported in three studies, irregular heart rates were reported in two studies, and no cardiac events were reported in two studies [38]. In general, MDA programs have not systematically monitored for cardiovascular adverse effects. With the popular off-label use of azithromycin combined with hydroxychloroquine early in the coronavirus disease 2019 (COVID-19) pandemic, there is evidence that this combination of drugs can lead to prolongation of QTc [45–47]. However, these studies frequently had a high proportion of adult participants with cardiac disease who were receiving other QT-prolonging agents. In several small observational studies, no QT prolongation was noted in hospitalized pediatric patients on hydroxychloroquine and azithromycin [48]. There remains a lack of evidence on the combined effect of hydroxychloroquine or chloroquine with azithromycin on the QT interval in non-hospitalized children. As malaria, HIV, and other conditions treated with QT-prolonging agents are present in many areas where MDA-azithromycin programs may be implemented, additive prolongation of the QT interval is a potential risk.

#### Chronic diseases

Antibacterial consumption has been linked to chronic diseases such as obesity, asthma, and inflammatory bowel disease through mechanisms that are thought to be related to alteration of the gut microbiome [49]. Among the antibacterials, macrolides in particular have been linked to the development of chronic disease in children. Among Finnish school children aged 2‒7 years who had used macrolides compared to those who had not used macrolides for > 2 years, there was a reduction in *Actinobacteria* composition and increase in *Bacteroidetes* and *Proteobacteria*, with reduction in microbial diversity that did not return to the level of control samples even 12‒24 months after the antibiotic course. In the group who used macrolides, there was a > 6 times odds of asthma compared to the non-exposed group. Similarly, those who received > 2 courses during the first 2 years were more likely to be overweight compared to the non-exposed group [49]. In retrospective birth cohorts in Canada, antibacterial exposure during the first year of life was associated with greater risk of asthma at 2‒9 years of age. Children who used macrolides (compared to other antibacterials) and those who used more courses of antibacterials, especially > 4 courses, had the highest risk of asthma [50]. An ecological study in the US and Europe showed that population-level consumption of macrolides was associated with higher risk of obesity [51].

### What are the antibacterial resistance risks associated with MDA-azithromycin?

The development of antibacterial resistance (ABR) in both targeted and commensal organisms is one of the greatest risks associated with MDA-azithromycin [6]. Unlike with helminths, where there is no evidence for sustained anti-helminthic drug resistance despite hundreds of millions of treatment courses with anti-parasitics, resistance in bacterial organisms is common [6]. In the 1960s, global attempts to eradicate malaria resulted in the emergence of drug resistance because of over-reliance on a single medication [1]. MDA programs for malaria are now only recommended in limited scenarios such as when elimination is near or in emergency outbreak settings, given both transient benefit and potential for drug resistance [52]. Similarly, there are many reports of rapid global dissemination of bacterial resistance, for example with the colistin resistance gene (*mcr-1*) and New Delhi metallo-beta-lactamase-1 gene (*NDM-1*), which render some of our broadest-spectrum antibacterials ineffective. Bacteria reproduce much more rapidly than parasites and can share resistance genes horizontally. Co-resistance, or resistance to more than one class of antibacterials that is often transmitted on mobile elements such as plasmids, and cross-resistance, in which a target used by multiple antibacterials is altered, can both result in resistance to multiple types of antibacterials [53]. With MDA-azithromycin, it is possible that the benefits may only be seen over a short period before ABR reaches levels that undermine any benefits of such programs [6] In addition, when macrolide resistance prevalence rises, macrolides can no longer be used reliably as empiric therapy for common infections such as pneumonia and diarrhea. In Table 4, we list the existing evidence on ABR following MDA-azithromycin, and we briefly summarize these findings below. The evidence suggests that the prevalence and duration of ABR following MDA-azithromycin varies depending on factors such as type of organism and number of rounds of MDA. Studies are increasingly moving from phenotypic to genotypic methods to identify macrolide resistance, which may also make comparisons between studies difficult.

**Table 4:** Evidence for antibacterial resistance (ABR) following MDA-azithromycin

Infection	Resistance evidence
Trachoma	•Three observational studies in Tanzania and Ethiopia assessed antibacterial resistance in *C. trachomatis* following one to four rounds of MDA-azithromycin. None of the studies found evidence of macrolide-resistant *C. trachomatis* 2–18 months after final treatment, although non-standard microbiological protocols were used in all studies [55]
Yaws	•In a region in Papua New Guinea where MDA-azithromycin was administered (with coverage of 84%) followed by targeted treatment programs, five cases of active yaws demonstrated clinical failure with azithromycin. Polymerase chain reaction (PCR) testing detected the A2059G mutation, a 23S rRNA mutation that can confer high-level resistance to macrolides and that is also found in *T. pallidum pallidum*. Interestingly, all clinical failures were detected among boys in the same village, thus the authors hypothesized that a de-novo mutation had arisen in one participant’s isolate and then been transmitted to others [54]
*Streptococcus pneumoniae*	•*S. pneumoniae* resistance to macrolides with MDA-azithromycin was documented as early as 1995 in children treated with a single dose of azithromycin for trachoma control, with 1.3% of children’s isolates showing resistance before and 21.3% of children’s isolates showing resistance up to 2 months after treatment [6] •A systematic review of studies through June 2018 found 19 studies exploring ABR after MDA-azithromycin, with 12 studies on *S. pneumoniae*. Baseline values of *S. pneumoniae* macrolide resistance prevalence in 6 studies ranged from 0 to 35.8%. Studies showed an increase in prevalence followed by a decrease, no increase after MDA-azithromycin, or no resistance at both time points [55] •Among children from Niger in MORDOR I, the proportion with *S. pneumoniae* macrolide resistance was higher in the MDA-azithromycin group (12.3%) compared to placebo (2.9%) after 4 rounds of treatment, indicating a four-fold rise [63] •In a subset of the Tanzanian group in MORDOR I, the proportion of macrolide-resistant *S. pneumoniae* isolates in children treated with azithromycin versus placebo at baseline (end of treatment), 12 months later, and 24 months later were 26.5%, 26.8%, and 13.4%, respectively, compared to 13‒18% in the placebo groups [59] •In a study exploring the impact of MDA-azithromycin with seasonal malaria chemoprophylaxis in Burkina Faso, there was increased *S. pneumoniae* resistance in several serotypes but resistance did not seem to favor one serotype in particular. Four serotypes 35B (2%), 23F (0.9%), 19F (0.9%), and 6A (0.7%) had the most macrolide-resistant isolates [90]
*Staphylococcus aureus*	•Three MDA-azithromycin rounds in the Gambia (PRET sub-study) were associated with a short-term increase in the prevalence of macrolide-resistant or macrolide-inducible, clindamycin-resistant *S. aureus*. Macrolide resistance was attributed to the presence of the *msr* and *erm* genes [56] Resistance dropped after cessation, but not to baseline levels [2]
*Streptococcus pyogenes*	•In a study of combined ivermectin and azithromycin compared to ivermectin alone in the Solomon Islands, there was no macrolide resistance detected in *S. pyogenes* at baseline, 3 months, or 12 months [57]
Enteric Organisms	•One RCT from Tanzania showed that a single round of MDA-azithromycin was associated with a substantial increase in macrolide-resistant *E. coli*, especially in younger children and those with prior diarrhea. Carriage prevalence decreased over time, but macrolide resistance remained elevated over baseline levels 6 months after dosing (from 21% at baseline to 61% at 1 month and 31% at 6 months) [58] •In a subset of the Tanzanian cohort from MORDOR I, at baseline, 12 months, and 24 months, the proportion of azithromycin-resistant *E. coli* isolates in the azithromycin arms increased transiently and then decreased (14.9%, 21.5%, and 14.9%, respectively, compared to 14–19% in the placebo arm) [59] •One study from Tanzania showed 17% of *E. coli* isolates from rectal swabs of children born after the last of 4 MDA-azithromycin rounds were resistant to azithromycin [91] •The Niger arm of MORDOR I showed determinants of macrolide resistance in the intestinal flora were higher in the MDA-azithromycin group (68.0%) compared to placebo (46.7%) at 6 months after 4 twice-yearly treatments with MDA [60] •Longer-term assessments among children in Niger were conducted while twice-yearly mass administration was occurring [61] Determinants of macrolide resistance remained higher in the azithromycin group compared to the placebo group: 7.4 times as high at 36 months and 7.5 times as high at 48 months

*MDA* mass drug administration, *MORDOR* Macrolides Oraux pour Réduire les Décès avec un Oeil sur la Résistance, *PRET* the Partnership for Rapid Elimination of Trachoma, *RCTs* cluster randomized trials, *TANA* Trachoma amelioration in Northern Amhara

#### Macrolide resistance in trachoma and yaws

While the development of ABR in organisms seems inevitable, no macrolide-resistant *C. trachomatis* serovars that cause trachoma have been documented to date in trachoma programs [5, 16]. Whether this finding is due to lack of resistance or lack of detection is unclear, as testing for antibacterial resistance in *C. trachomatis* is not widespread. Similarly, for many years, macrolide resistance in *T. pallidum pertenue* was not detected following MDA-azithromycin. However, a more recent study from Papua New Guinea described macrolide-resistant isolates *T. pallidum pertenue* in communities where MDA-azithromycin had been used for many years [54].

#### Macrolide resistance in gram-positive bacteria: *Streptococcus pneumoniae*, *S. aureus*, and *S. pyogenes*

Many studies have explored the development of macrolide resistance in *S. pneumoniae* following MDA-azithromycin. Based on data from observational and interventional studies, it appears that in general, risk of macrolide resistance in *S. pneumoniae* (1) increases with increased rounds of MDA, (2) is greater when there is higher baseline burden of macrolide-resistant *S. pneumoniae*, (3) decreases with the cessation of MDA-azithromycin, but may not return to baseline levels, and (4) does not appear to be related to *S. pneumoniae* serotype [55] However, the overall body of data regarding *S. pneumoniae* macrolide resistance with MDA-azithromycin are conflicting.

Data regarding macrolide resistance in *S. aureus* indicate that resistance increases following MDA-azithromycin, and then decreases following cessation of MDA-azithromycin. However, studies are limited [2, 56]. No macrolide resistance has been detected in *S. pyogenes* following MDA-azithromycin, although studies again are limited [57].

#### Macrolide resistance in enteric organisms and gut microbiota

Available data indicate that MDA-azithromycin is associated with an increase in antibacterial resistance in enteric organisms. Two studies using phenotypic testing for resistance from Tanzania showed increased macrolide-resistant *E. coli* which persisted for the six months of observation in one study and over at least 12 months in the other [58, 59]. In the Niger arm of MORDOR I, where genotypic testing was conducted, determinants of macrolide resistance in intestinal flora were elevated at six months in one study and persisted through 48 months in another [60, 61]. Such increases are concerning, although not surprising, since gram-negative organisms can develop antibacterial resistance rapidly and are able to easily spread resistance elements via horizontal gene transfer of mobile elements.

#### Macrolide cross-resistance with other antibiotics

Both cross-resistance and co-resistance with other antibacterials could pose problems when considering MDA-azithromycin implementation. Azithromycin could be a potent driver of cross-resistance because of its long half-life, high intracellular tissue concentration, and large volume of distribution. However, studies to date are limited and provide conflicting evidence regarding cross-resistance with other classes of antibacterials, with the exception of the lincosamide clindamycin, to which cross-resistance is common. The *erm* genes are responsible for the most widespread form of macrolide resistance (known as the MLS_B_ phenotype), which results in cross-resistance between macrolides, lincosamides like clindamycin, and streptogramin B [62].

The Niger arm of MORDOR I showed a significant difference in clindamycin-resistant *S. pneumoniae* in MDA-azithromycin versus placebo groups [2, 63] However, the proportion of *S. pneumoniae* with resistance to penicillin was similar in the MDA and placebo groups at 24 months, and there were no significant differences in non-macrolide resistance determinants in either nasopharyngeal or rectal swabs between the two groups [60, 63] Interestingly, at 36 months while twice-yearly mass administration was still occurring, MDA-azithromycin selected for non-macrolide resistance determinants, including to beta-lactam antibacterials (2.13 factor difference), tetracyclines (1.68), trimethoprim (2.22), and aminoglycosides (2.32 factor difference). However, only the macrolide, beta lactam, and tetracycline resistance determinants stayed elevated at 48 months [61] The authors note that all but tetracycline belong to the WHO Access group of antibiotics that are typically used against a wide range of community-acquired infections, raising concern if these antibiotics become ineffective.

### Summary and considerations when implementing MDA-azithromycin programs

Over the past decade, MDA-azithromycin programs have gained increasing attention due to positive impacts beyond their original intent. Available evidence suggests that MDA-azithromycin is beneficial in reducing mortality in children < 5 years of age, combatting the NTDs trachoma and yaws, and possibly reducing the burden of common infectious diseases such as diarrheal illness and impetigo. Interventions that target multiple diseases simultaneously tend to be more cost-effective, and MDA-azithromycin has the potential to affect multiple conditions (NTDs, high child mortality) that occur in the same region [64].

Many questions remain regarding the appropriate use of MDA-azithromycin. The regions and populations in which MDA-azithromycin may provide most benefit must be better understood. It would be prudent for policymakers considering MDA-azithromycin to map out geographical areas in which the highest burden of under-five mortality, targeted NTDs, and illnesses such as ARI, diarrheal illness, and malaria exist. The optimal age group to be targeted must also be determined. The WHO recommends MDA-azithromycin in children < 1 year of age for reducing under-five mortality, since the greatest reduction in mortality occurred in this age group. However, studies suggest that limiting the intervention to those 1‒5 months old or 1‒11 months old would reduce the number of deaths averted by 2.5 to sixfold, given the larger population size in the older age groups, and may decrease the potential positive impact of MDA-azithromycin [65].

The optimal dose, frequency, and number of intervention cycles of MDA-azithromycin has also not been determined yet, and further studies are needed to answer these questions. Local acceptability and preferences must ultimately be given greatest consideration when deciding whether to implement MDA-azithromycin. Local populations may consider the short-term benefits of reduced child mortality to far outweigh longer-term, theoretical risks such as ABR. However, with antimicrobial resistance forecasted to cause millions of deaths and dollars lost in economic productivity by 2050, the benefits and risks associated with MDA-azithromycin programs must be balanced carefully [66]. The lack of a clear rationale by which MDA-azithromycin programs decrease under-five mortality may give policymakers some pause. Evidence to date has not supported a decrease in infectious disease burden as driving the reduction in under-five mortality, although this mechanism is considered most likely. Changes such as modulation of inflammation or increases in weight are interesting hypotheses that need to be explored in future studies.

When implementing MDA-azithromycin programs, attention needs to be given to systematically monitoring adverse effects and antibacterial resistance. Historically, MDA-azithromycin programs have not conducted thorough assessments of mild adverse reactions such as gastrointestinal side effects or even more severe adverse reactions such as IHPS. In MORDOR I, parents were instructed to monitor for adverse effects and report them to a village representative, and such a strategy could be utilized in the future. The impact of MDA-azithromycin on childhood chronic diseases, such as asthma and obesity, has also not been assessed to date. Monitoring these diseases is critical, as LMICs could subsequently face a greater dual burden of communicable and non-communicable diseases [67]. The WHO launched the Global Antimicrobial Resistance Surveillance System (GLASS) in 2015 to support global surveillance to strengthen the evidence base on antimicrobial resistance [68]. GLASS could be leveraged for improving ABR surveillance following MDA-azithromycin. As genomic approaches become more routine and less expensive, combining phenotypic and genotypic approaches to monitor AMR may help generate a larger volume of useful data [61]. Given the growing threat of ABR on health, ABR assessments should include baseline resistance testing followed by multiple cross-sectional assessments, with household contacts also being investigated to generate a broader understanding of ABR risk [69]. Many of the communities that stand to benefit the most from MDA-azithromycin do not have the infrastructure in place to conduct surveillance for adverse effects and ABR, prerequisites recommended by the WHO prior to implementing MDA-azithromycin. Communities where MDA-azithromycin should and can practicably be implemented need to be identified.

## Conclusions

MDA-azithromycin programs have shown significant clinical benefits as well as potential risks that should be weighed carefully when deciding how, when, and where to best implement such programs. Impact at the individual, community, and global levels in both the near and long terms should be considered when making policy decisions regarding MDA-azithromycin implementation.

## Data Availability

Not applicable.

## Abbreviations

ABR: Antibacterial resistance
ARI: Acute respiratory infection
CDC: Centers for Disease Control and Prevention
GET2020: Global Elimination of Trachoma by 2020
IHPS: Idiopathic hypertrophic pyloric stenosis
IM: Intramuscular
LMICs: Low- or middle-income countries
LRTI: Lower respiratory tract infection
MDA: Mass drug administration
MORDOR: Macrolides Oraux pour Réduire les Décès avec un Oeil sur la Résistance
NTDs: Neglected tropical diseases
RCTs: Cluster randomized trials
SDG: Sustainable development goal
SMC: Seasonal malaria chemoprophylaxis
TANA: Trachoma amelioration in Northern Amhara
WHO: World Health Organization
